# Cardiovascular Factors Associated with Septic Shock Mortality Risks

**DOI:** 10.51894/001c.6516

**Published:** 2018-04-27

**Authors:** Jelena Arnautovic, Areej Mazhar, Britni Souther, Gary Mikhijan, J. Boura, Najia Huda

**Affiliations:** 1 St. John Macomb, Department of Cardiovascular Medicine, Faculty, Warren MI; 2 Henry Ford Macomb, Department of Internal Medicine, PGY 3 Resident, Clinton Township, MI; 3 Henry Ford Macomb, Department of Emergency Medicine, PGY1 Resident, Clinton Township, MI; 4 St. John Macomb, Department of Cardiovascular Medicine, Statistician, Warren, MI; 5 Henry Ford, Department of Critical Care Medicine, Attending Faculty, Detroit, MI

**Keywords:** mortality risks, cardiovascular disease, septic shock mortality

## Abstract

**CONTEXT:**

The presence of at least one underlying chronic health condition, such as long-term care facility residence, malnutrition, immunosuppression, or prosthetic device use, are well known factors increasing infection risks and progression to severe sepsis. Furthermore, some degree of cardiovascular dysfunction occurs in the majority of septic patients and this prognostic significance has become increasingly recognized. Since septic shock carries the highest mortality risk on the sepsis spectrum, it is important to evaluate the cardiovascular risk impact on mortality in this subset of patients.

**METHODS:**

The retrospective parent study contributing these electronic health record data was IRB approved and conducted across four hospital intensive care units within the authors’ Michigan healthcare system. Patients with cardiopulmonary arrest or transfers from an outside facility were excluded. The authors evaluated the presence of modifiable and non-modifiable cardiovascular risk factors in septic shock patients upon admission to an emergency department.

**RESULTS:**

The authors’ final analytic sample included n = 109 adults who were discharged alive compared to those who died during hospitalization. Those patients who died were more often male with an underlying history of hypertension, congestive heart failure, coronary artery disease, or peripheral arterial diseases, were taking pre-admission beta-blocker medications, and had higher APACHE II scores at admission compared to the patients who survived to discharge. Significantly higher mortality risks were found in sample patients with increased troponin levels on admission and atrial fibrillation.

**CONCLUSIONS:**

Appropriate triage and prompt treatment of these patient groups with tailored therapy to stabilize and improve cardiac dysfunction in the emergency department could potentially lead to improved survival outcomes. Clinicians need more studies to determine therapeutic targets most impacting underlying pathophysiologic mechanisms such as elevated troponin and atrial fibrillation that greatly increase mortality risks.

## INTRODUCTION

Sepsis can be a potentially life-threatening complication of infection. Sepsis occurs when chemicals released into the bloodstream normally to fight infection trigger inflammatory responses throughout the body.^1^ This inflammation inadvertently triggers a cascade of changes that can damage multiple organ systems, causing them to fail.^1^ The incidence of severe sepsis increases disproportionately in older adults, with more than half of severe sepsis cases occurring in adults over 65 years of age.^2^ The majority of severe sepsis patients also have at least one chronic health condition such as chronic obstructive pulmonary disease, malignancy, chronic renal or liver disease, and diabetes.^3^ Other risk factors for progressing to severe sepsis include living in a long-term care facility, malnutrition, immunosuppressive therapy, and using a prosthetic device.^3^

A compromised immune system will significantly increase a patient’s risk for infection and severe sepsis.^3^ Some degree of cardiovascular dysfunction is common in patients who become critically ill with sepsis, and the incidence increases with the severity of their cardiac impairment.^4^ Severe sepsis occurs in nearly 70% of all septic patients and can manifest as hemodynamic instability, cardiac biomarker elevation, myocardial dysfunction on echocardiography, and end‐organ hypo-perfusion.^4^

Cardiovascular dysfunction in sepsis is associated with relatively worse hospital and long‐term outcomes, necessitating early diagnosis and management.^5^ The severity of a patient’s infection and initiation of proper management protocols often guides providers’ use of clinical data applied to the sepsis criteria. Some non-modifiable risk factors in conjunction with modifiable risk factors can place certain septic shock patients at a higher risk for death, and it is thus imperative to modify sepsis therapy accordingly.

The primary goals of these retrospective analyses were to evaluate the presence of modifiable and non-modifiable cardiovascular risk factors in septic shock patients upon admission to an emergency department (ED) and investigate the impact of specific cardiovascular risk factors on mortality in septic shock patients.

## METHODS

### Study design

Before data collection, the Institutional Review Board at the Henry Ford Hospital, Detroit, Michigan, had approved the parent study from which these data were derived. The authors’ study population included a historical cohort of all consecutive adults admitted for septic shock over a 32-month period. The study had been conducted across four Henry Ford Health System university-affiliated hospital intensive care units.

### Sample Patients

The analytic sample was comprised of the population of non-pregnant adult patients aged 18 to 90 years old diagnosed with septic shock within the first 48 hours of their hospital admission. For these analyses, septic shock was defined as patients requiring vasopressors to maintain a mean arterial pressure of 65 mm. Hg. or greater despite adequate fluid resuscitation, as well as a serum lactate level greater than 2.0 mmol/L in the absence of hypovolemia.^6^ Patients who had sustained a cardiopulmonary arrest or transferred from another facility were excluded. Overall, 719 septic shock patients were initially screened, and an analytic convenience sample of n = 109 (15.2%) eligible patients met inclusion criteria.

### Data Collection and Definitions

The electronic medical records of eligible patients diagnosed with septic shock were reviewed, and data pertaining to their socio-demographics and pertinent comorbid conditions (i.e., cardiovascular conditions) were sought. Over two-to-three months, the team of authors manually extracted data concerning the impact of cardiovascular risk factors on mortality outcomes. Of note, prior and during hospitalization transthoracic echocardiographic (TTE) data were evaluated. However, this sample subgroup was small and reporting was not consistent (i.e., some patients only had received a limited TTE). Therefore, only ejection fraction data prior and during patients’ hospitalization was reported. Patients’ past history of hypertension (HTN) and previous antihypertensive medication use was reviewed, specifically beta-blockers and calcium channel blockers.

Patients’ biologic profile, including hemoglobin, albumin, cardiac biomarkers, and their overall impact on mortality was also evaluated. Troponin labs were performed using the Siemens Centaur Method (TnI) with results >99th percentile of the upper reference limit (0.05 ng/mL correlating with myocardial injury) in association with clinical signs and electrocardiographic (EKG) findings. Data concerning the characteristics and source of the primary infection were also obtained. Mortality risk at admission was assessed using the Acute Physiological and Chronic Health Evaluation II (APACHE II) scoring method.^7^ The well-validated modified form of the Charlson Comorbidity Index method ^8^ was also used to stratify patients by composite comorbidity on the basis of their documented healthcare conditions.

### Statistical Analysis

All analyses were performed by author JB using SAS for Windows ® 9.4.^9^ No missing data were replaced by substitutions or interpolations. Depending on the normality distribution of data, all continuous data were described using means and standard deviations, or medians and 25^th^, 75^th^ percentiles. Categorical data were described using counts and percent frequencies. Patients who survived to discharge were compared to those who died during their hospital stay. Continuous variables were examined using Wilcoxon rank sum tests. Categorical variables were examined with Chi-square tests where appropriate (expected frequency > 5 in 80% of cells), otherwise Fisher’s Exact tests were used. Odds ratios (OR) and 95% confidence intervals (CI) were calculated where possible.

## RESULTS

During the analytic period, a total of n = 109 patients met our inclusion/exclusion criteria. 48 (44.0%) patients were male and 61 (56.0%) were female, and their predominant reported race was Caucasian 79 (72.5%). Patients’ mean age was 68 years (SD 13.6). As far as pre-existing cardiovascular diseases, 66 (60.5%) of the patients had a history of HTN, 89 (81.7%) had known history of congestive heart failure (CHF), and 53 (48.6%) had atrial fibrillation (AF). Coronary artery disease (CAD) was also reported in 35 (32.1%) of the patients.

The majority of patients reported tobacco use, 58 (59.2%). In regards to cardiac biomarkers, troponin was elevated in 58 (55.8%) of patients on admission, and the median brain natriuretic peptide (BNP) in the first 24 hours was 389 pg/ml (25^th^ = 157, 75^th^ = 753). A subgroup of 63 (57.8%) patients had a prior TTE on record and 53 (84.1%) of these patients had preserved left ventricular ejection fraction (LVEF). LVEF during hospitalization was preserved in 57 (71.3%) of the 80 patients with a value reported. Median intensive care unit length of stay was five days, and median hospital stay was eight days. Mortality was quite high with 55 (50.5%) patients dying during hospitalization and 18 out of 54 (33.3%) readmitted within one month. Ten (59.6%) more sample patients died by three months and an additional one patient died at 12 months, total 60.6%. (Table 1)

**Table 1:** Descriptive Characteristics of Sample Patients (N=109)

**Variable**	**N (%)**
Male	48 (44.0%)
**Race**	N=103
White	79 (76.7%)
Black	24 (23.3%)
**Age**	
Mean ± SD (median)	68 ± 14 (69)
Min to max	18 to 89
BMI*	
Median (25 ^th^, 75 ^th^)	27.4 (23.0, 32.8)
Min to Max	13 to 102
HTN *	66 (60.6%)
Any CHF*	89 (81.7%)
CAD*	35 (32.1%)
MI*	16 (14.7%)
Peripheral diseases	11/99 (11.1%)
Diabetes (any form)	33 (30.3%)
CVA*	23 (21.3%)
COPD*	43 (39.5%)
CKD III*	32 (29.4%)
ESRD	10 (9.2%)
Pre-Admission Beta Blocker	49 (45.0%)
Pre-Admission Calcium Channel Blockers	12 (19.3%)
Tobacco use	58/98 (59.2%)
SI resp*	56 (51.4%)
SI GI	18 (16.5%)
SI GU	29 (26.6%)
SI bone	3 (2.8%)
SI skin	13 (11.9%)
SI unclear	5 (4.6%)
SI blood	19 (17.4%)
Any AF *	53 (48.6%)
Serum albumin < 3.5 g/dl	97 (89.0%)
Admission hemoglobin <10	45 (41.3%)
Troponin elevated in the first 24 hours	58/104 (55.8%)
**Peak troponin 24h**	N=104
Median (25 ^th^, 75 ^th^)	0.07 (0.04,0.50)
Min to Max	0.04 to 25.46
Admission ventricular response>110	50 (45.9%)
EF* prior to hospitalization	N=63
<35%	6 (9.5%)
35-45%	4 (6.4%)
>45%	53 (84.1%)
**EF during hospitalization**	N=80
<35%	15 (18.8%)
35-45%	8 (10.0%)
>45%	57 (71.3%)
**Valvulopathy**	N=41
Mild	14 (34.1%)
Moderate	23 (56.1%)
Severe	4 (9.8%)
Mechanical ventilation	73 (67.0%)
BNP*	N=64
Median (25 ^th^, 75 ^th^)	389 (157, 753)
Min to Max	18 to 8000
APACHE II* ^6^	
Median (25 ^th^, 75 ^th^)	20 (15, 26)
Min to Max	8 to 45
**Charlson Index (age adjusted) ^7^**	
Median (25 ^th^, 75 ^th^)	7 (4, 9)
Min to Max	0 to 19
ICU* length of stay (days)	
Median (25 ^th^, 75 ^th^)	5 s(3, 10)
Min to Max	1 to 32
**Hospital length of stay (days)**	
Median (25 ^th^, 75 ^th^)	8 (4, 13)
Min to Ma	1 to 35
In-hospital death	55 (50.5%)
Died within three months	65 (59.6%)
Died within one year	66 (60.6%)
Readmission within one month	18/54 (33.3%)

*BMI-body mass index, CHF-congestive heart failure, CAD-coronary artery disease, MI-myocardial infarction, CVA-cerebrovascular accident, COPD-chronic obstructive pulmonary disease, CKD-chronic kidney disease, ESRD-end-stage renal disease, SI-source of infection, GI-gastrointestinal, GU-genitourinary, EF-ejection fraction, BNP-brain natriuretic peptide, APACHE-acute physiology and chronic health evaluation, ICU-intensive care unit.

Furthermore, bivariate associations of selected study measures with in-hospital mortality were performed. The presence of underlying cardiovascular diseases had a significant impact on mortality in these septic shock patients. Patients who died were more often male (p = 0.003), hypertensive (p = 0.043), had coronary (p = 0.029) and peripheral artery disease (p = 0.028). Additionally outpatient beta-blocker use (p = 0.016), any AF (p = 0.017), troponin elevation in first 24 hours (p = 0.009) troponin peak at first 24 hours (p = 0.003), and invasive mechanical ventilation (p = 0.002) were significant in patients who died. Also notable, there was an increased incidence of respiratory tract infections in this patient sample, although the highest mortality was associated with gastrointestinal infections (p = 0.011) and skin infections had the lowest mortality (p = 0.007). APACHE II scores from admission were also compared between those patients with in-hospital mortality to those patients who survived to discharge. (Tables 2 and 3)

**Table 2:** Bivariate Associations with In-hospital Mortality**

	**In-hospital Survival**	**In-Hospital Mortality**	**P-value**	**OR (95% CI)**
**Variable**	**(N= 54)**	**(N= 55)**		
Male	16 (29.6%)	32 (58.2%)	**0.003**	3.30 (1.50, 7.30)
Race	N=52	N=51		
White	37 (71.2%)	42 (82.4%)	0.18	0.53 (0.21, 1.35)
Black	15 (28.9%)	9 (17.7%)		
Age				1.39 (1.03, 1.87) in increments of 10
Mean ± SD (median)	65 ± 15 (66)	71 ± 11 (71)	0.061	
Min to max	18 to 89	46 to 88		
BMI*				
Median (25^th^, 75^th^)	28.7 (21, 36)	26.7 (24, 32)	0.48	0.97 (0.93, 1.01)
Min to Max	13 to 102	17 to 54		
HTN*	26 (48.2%)	40 (72.7%)	**0.009**	2.87 (1.29, 6.38)
Any CHF*	40 (74.1%)	49 (89.1%)	**0.043**	2.86 (1.01, 8.12)
CAD*	12 (22.2%)	23 (41.8%)	0.029	2.52 (1.09, 5.80)
MI*	6 (11.1%)	10 (18.2%)	0.3	1.78 (0.60, 5.29)
Peripheral diseases	2/49 (4.1%)	9/50 (18%)	**0.028**	5.16 (1.05, 25.3)
Diabetes (any)	15 (27.8%)	18 (32.7%)	0.57	1.26 (0.56, 2.87)
CVA*	9 (16.7%)	14/54 (25.9%)	0.24	1.75 (0.68, 4.48)
Outpatient beta blocker	18 (33.3%)	31 (56.4%)	**0.016**	2.58 (1.19, 5.62)
Any AF*	20 (37.0%)	33 (60%)	**0.017**	2.55 (1.18, 5.52)
Tobacco	27/49 (55.1%)	31/49 (63.3%)	0.41	1.40 (0.63, 3.15)
SI* resp	26 (48.2%)	30 (54.6%)	0.5	1.29 (0.61, 2.74)
SI GI*	4 (7.4%)	14 (25.5%)	**0.011**	4.27 (1.30, 14.0)
SI GU*	16 (29.6%)	13 (23.6%)	0.48	0.74 (0.31, 1.73)
SI bone	1 (1.9%)	2 (3.6%)	1	2.00 (0.18, 22.7)
SI skin	11 (20.4%)	2 (3.6%)	**0.007**	0.15 (0.03, 0.70)
SI unclear	3 (5.6%)	2 (3.6%)	0.68	0.64 (0.10, 4.00)
SI blood	8 (14.8%)	11 (20.0%)	0.48	1.44 (0.53, 3.91)
Serum albumin < 3.5 g/dl	46 (85.2%)	51 (92.7%)	0.21	2.22 (0.63, 7.85)
Admission hemoglobin <10	22 (40.7%)	23 (41.8%)	0.91	1.05 (0.49, 2.24)
Troponin elevated in the first 24 hours	20/51 (39.2%)	38/53 (71.7%)	**0.0009**	3.93 (1.73, 8.92)
Peak troponin 24h	N=51	N=53	**0.0003**	NA
Median (25^th^, 75^th^)	0.04(.04,.10)	0.21(.04,1.72)		
Min to Max	0.04 to 25.46	0.04 to 100		
Admission ventricular response>110	26 (48.2%)	24 (43.6%)	0.64	0.83 (0.39, 1.77)
EF* during hospitalization	N=37	N=43	0.054	NA
<35%	3 (8.1%)	12 (27.9%)		
35-45%	3 (8.1%)	5 (11.6%)		
>45%	31 (83.8%)	26 (60.5%)		
Mechanical ventilation	27 (50.0%)	46 (83.6%)	**0.0002**	5.11 (2.10, 12.5)

*BMI-body mass index, HTN-hypertension, CHF-congestive heart failure, CAD-coronary artery disease, MI-myocardial infarction, CVA-cerebrovascular accident, AF-atrial fibrillation, SI-source of infection, GI-gastrointestinal, GU-genitourinary, EF-ejection fraction

**Please direct any and all questions regarding table specifics to the corresponding author

**Table 3:** Continuous Variables comparing Patients Surviving to Discharge to those Dying In-hospital.

**Variable**	**In-hospital** **Survival** **(N= 54)**	**In hospital** **Mortality** **(N= 55)**	**P-value**
Peak lactic acid/24hmmol/L Median (25^th^, 75^th^) Min to Max	2.9 (1.8, 4.4) 0.3 to 21	3.6 (2.0, 5.8) 0.7 to 22.5	0.21
BNP* peak 24h (pg/ml) Median (25^th^, 75^th^) Min to Max	N=27 241 (79, 747) 18 to 4000	N=37 478 (260, 1222) 20 to 8000	0.10
APACHE II * ^6^ Median (25^th^, 75^th^) Min to Max	18 (14, 24) 8 to 38	21 (17, 28) 10 to 45	**0.009**
Charlson Comorbidity Score ^7^ Median (25^th^, 75^th^) Min to Max	6.5 (4, 9) 0 to 19	8.0 (5, 10) 0 to 15	0.07

*BNP-brain natriuretic peptide,

APACHE-acute physiology and chronic health evaluation

## DISCUSSION

Based on these results and those from earlier studies, cardiovascular compromise is a common occurrence in critically ill patients with sepsis, with its incidence rising with severity of illness, increasing age, and the increasing number of comorbidities.^4,5^ In these analyses, markers of illness severity such as multi-organ failure and hypo-perfusion and several critical care interventions (e.g., intravenous vasopressors, fluid resuscitation) were also associated with an increased in-hospital mortality.

Additionally, in this analytic sample septic shock patients with a prior cardiovascular history of HTN, CAD, CHF, AF and peripheral arterial disease had increased in-hospital mortality (p < 0.05). Those patients with a reduced LVEF had higher mortality risks, although not at statistically significant levels (p = 0.054). Based on prior literature, arrhythmias are commonly encountered by physicians in septic shock patients as a pathophysiological compensatory response.^10^ In our analyses, overall in-hospital mortality risks were significantly influenced by presence of any type of AF. In 2014, Kuipers et al. reported that even the occurrence of a single episode of AF was associated with increased mortality, increased length of stay and a possibly increased risk of stroke in sample patients.^10^

Our team found that the majority of patients had elevated troponin (TnI) on admission. It has been established that cardiac troponin levels correlate with the presence of left and right ventricular dysfunction on echocardiography as well as higher hospital mortality.^11–14^ About 85% of patients with sepsis and septic shock have detectable cardiac troponin levels using standard troponin assays.^12–15^ In several studies, however, troponin levels have demonstrated a variable association with mortality, as well as a correlation with the duration of hypotension and extent of vasopressor support.^16–18^

In patients with sepsis and septic shock, physicians commonly see an elevated Tnl level associated with increased prevalence of CAD and higher illness severity. After controlling for other clinical factors, elevations in Tnl were independently associated with increased short and long‐term mortality risks.^19^ In our sample of septic shock patients, we did demonstrate that elevated TnI values correlated with poor prognosis and higher mortality risks (p < 0.01).

Despite its increasingly frequent recognition, the etio-pathogenesis of troponin elevation in sepsis remains uncertain, and EKG and echocardiography in such patients rarely demonstrate ischemic changes, inducible ischemia or occlusive coronary thrombus on further stress testing or invasive testing (e.g., cardiac catheterization).^12,20^ Flow‐limiting CAD is infrequently documented in these patients, alluding to alternate mechanisms of Tnl other than an acute ischemic event.^12^

Postulated causes for troponin elevations in septic patients include ischemic mechanisms similar to those with ischemic hepatopathy. These mechanisms include supply demand mismatch or microvascular thrombus, as well as non-ischemic mechanisms such as reversible myocardial membrane leakage of cytosolic TnT pool or direct cellular toxicity from inflammatory mediators or excessive catecholamine levels.^21–23^

These analytic results demonstrate that the in-hospital mortality of septic shock patients was higher in those with prior cardiovascular history including HTN, obstructive CAD, peripheral artery disease, and underlying arrhythmias such as AF. In our analyses, patients’ mortality risks were significantly associated with elevated TnI levels, demonstrating the value and pertinence of carefully monitoring their TnI levels and recognizing non-ischemic etiologies of their elevation.

Additionally, these results demonstrate that lactic acid elevation (which is commonly used as a predictor of shock and poor outcome in shock) was not as statistically significant as troponin elevation and troponin peak in the first 24 hours (P = 0.210). This finding brings into question whether there can be a utility in sepsis tool predictors using troponin values in addition to lactic acid levels.

Our analyses have several limitations in that they were conducted with retrospective data and our sample was limited to a convenience sample of hospitalized septic shock patients in a single healthcare system. We acknowledge that we may have been inadequately powered to detect additional significant associations that might have been found from a larger sample. It is important to note that these analyses used data derived from a larger parent study that the authors are confident will lead to additional papers concerning the impact of new onset of AF in septic shock patients.

## CONCLUSIONS

Troponin levels and other markers of illness severity may be triggered by profound inflammatory response particularly in patients with underlying cardiovascular disease. Elevated troponins tend to be associated with increased risk of both short and long‐term mortality. Our study findings indicate that patients with history of underlying CAD and HTN who present with septic shock within the first 48 hours of admission and have Tnl and other markers of inflammatory and ischemic response such as atrial fibrillation tend to have a higher mortality.

Appropriate triage and recognition of this subset of patients along with tailoring therapy to stabilize and improve cardiac dysfunction and resuscitation can potentially lead to better outcomes. Although the results from prior studies agree with this conclusion, few studies have examined outcomes of patients who receive early resuscitation with AF and elevated troponins. Further studies focusing on adequate resuscitation within the setting of elevated troponins are certainly required. These study findings further affirm the need for an improved understanding of the underlying etio-pathogenesis and mechanisms during critical sepsis illness to impact patient outcomes and mortality.

### Conflict of Interest

The authors declare no conflicts of interest.
